# Taxonomy and phylogeny of brown-rot corticioid fungi in China: *Coniophora beijingensis* and *Veluticeps subfasciculata* spp. nov.

**DOI:** 10.3389/fmicb.2023.1133236

**Published:** 2023-03-16

**Authors:** Yue Li, Shuang-Hui He

**Affiliations:** School of Ecology and Nature Conservation, Beijing Forestry University, Beijing, China

**Keywords:** Coniophoraceae, Gloeophyllaceae, species diversity, taxonomy, wood-decaying

## Abstract

Brown-rot fungi account for a small portion of the wood-decaying fungi. There are a few corticioid genera causing brown rot of wood, and their species diversity is still under investigated and studied, especially in subtropical and tropical areas. Two new brown-rot corticioid fungi, *Coniophora beijingensis* and *Veluticeps subfasciculata* were found during the investigation of corticioid fungi in China. Phylogenetic analyses of the two genera were carried out separately based on ITS-28S sequence data. *Coniophora beijingensis* was collected from Beijing, north China, from different kinds of angiosperm and gymnosperm trees, and is characterized by possessing a monomitic hyphal system with colorless hyphae and relatively small pale yellow basidiospores 7–8.6 μm× 4.5–6 μm. *Veluticeps subfasciculata* was collected from Guizhou and Sichuan Provinces, southwestern China, on *Cupressus* and is characterized by the resupinate to effused-reflexed basidiomes with a colliculose hymenophore, nodose-septate generative hyphae, fasciculate skeletocystidia and subcylindrical to subfusiform basidiospores 8–11 μm × 2.5–3.5 μm. Descriptions and illustrations are provided for the two new species, and identification keys to *Coniophora* and *Veluticeps* species in China are given. *Coniophora fusispora* is reported in China for the first time.

## Introduction

Modern phylogenetic analyses indicated that corticioid brown-rot fungi distributed in several main lineages of Agaricomycetes, such as, Boletales, Polyporales, Gloeophyllales, and Amylocorticiales (Larsson, 2007; Hibbett et al., 2014). The total number of this group of fungi is small with some relatively small but important genera, among which *Coniophora* DC. and *Veluticeps* Cooke are two typical ones.

*Coniophora* is an old genus and includes 170 names (Index of Fungorum)^1^ but many of them are synonyms (Ginns, 1982). Morphologically, species of *Coniophora* are characterized by having resupinate basidiomes with a smooth to tuberculate hymenophore, usually simple-septate (occasionally nodose-septate) generative hyphae, thick-walled, pale yellow to brown basidiospores with dextrinoid and cyanophilous reactions, and lacking cystidia (present in one species, Ginns, 1982; Bernicchia and Gorjón, 2010). *Leucogyrophana* Pouzar and *Serpula* (Pers.) Gray are two similar genera but differ in having merulioid to irpicoid hymenophores and nodose-septate generative hyphae (Bernicchia and Gorjón, 2010). Phylogenetically, *Coniophora* formed a well-supported lineage in Boletales and was closely related to *Coniophoropsis* Hjortstam & Ryvarden, which have distinct ornamented basidiospores (Hjortstam and Ryvarden, 1986; Zhao et al., 2018).

*Veluticeps* Cooke, typified by *V. berkeleyana* Cooke, is characterized by the resupinate, effused-reflexed or pileate basidiomes with a smooth, warted or odontioid hymenophore, a monomitic hyphal system with simple- or nodose-septate hyphae, single or fasciculate cystidia and ellipsoid or cylindrical basidiospores negative in Melzer’s reagent (Nakasone, 1990; Bernicchia and Gorjón, 2010). Phylogenetic studies indicated that *Veluticeps* is a member of the small order Gloeophyllales, which include the famous brown-rot poroid genus, *Gloeophyllum* P. Karst. (Larsson, 2007; Garcia-Sandoval et al., 2011; He and Li, 2013; Yang et al., 2016). Nakasone (1990, 2004) did monographic studies on the genus and other similar genera. The genus now comprises about 11 species with several new species were described from China based on morphological and molecular evidence recently (He and Li, 2013; Yang et al., 2016; Yang and He, 2016).

Although the poroid brown-rot fungi in China have been intensively studied in recent years (Han et al., 2016; Shen et al., 2019; Liu et al., 2021), the species diversity of the corticioid ones are still largely unknown. Preliminary morphological and molecular studies on the corticioid specimens collected from China recently revealed two undescribed species. In this paper, we carried out two independent phylogenetic analyses based on ITS-28S sequence data, and describe and illustrate the two new species as *Coniophora beijingensis* and *Veluticeps subfasciculata*.

## Materials and methods

### Specimen collection

*In situ* photos of specimens were taken with a Canon camera EOS 70D (Canon Corporation, Japan). Specimens were dried with a portable dryer, labelled, then stored in a freezer at minus 40°C for 2 weeks to kill the insects and their eggs before proceeding with morphological and molecular studies. Voucher specimens are deposited at the herbarium of Beijing Forestry University, Beijing, China (BJFC). Herbarium code designations follow Index Herbarium.^2^

### Morphological studies

Thin, freehand sections were made from dried basidiomes and mounted in 2% (weight/volume) aqueous potassium hydroxide (KOH) and 1% (w/v) aqueous phloxine. Amyloidity and dextrinoidity of hyphae and basidiospores were checked in Melzer’s reagent (IKI). Cyanophily of hyphal and basidiospore walls was observed in 1% (w/v) cotton blue in 60% (w/v) lactic acid (CB). Microscopic examinations were carried out with a Nikon Eclipse 80i microscope (Nikon Corporation, Japan) at magnifications up to 1,000 ×. Drawings were made with the aid of a drawing tube. The following abbreviations are used: L = mean basidiospore length, W = mean basidiospore width, Q = L/W ratio, n (a/b) = number of basidiospores (a) measured from number of specimens (b). Color codes and names follow Kornerup and Wanscher (1978).

### DNA extraction and sequencing

A CTAB plant genomic DNA extraction Kit DN14 (Aidlab Biotechnologies Co., Ltd., Beijing, China) was used to extract total genomic DNA from dried specimens then amplified by the polymerase chain reaction (PCR), according to the manufacturer’s instructions. The ITS1-5.8S-ITS2 region (ITS) was amplified with the primer pair ITS5/ITS4 (White et al., 1990) using the following protocol: initial denaturation at 95°C for 4 min, followed by 34 cycles at 94°C for 40 s, 58°C for 45 s and 72°C for 1 min, and final extension at 72°C for 10 min. The nrLSU D1-D2 region (28S) was amplified with the primer pair LR0R/LR7^3^ employing the following procedure: initial denaturation at 94°C for 1 min, followed by 34 cycles at 94°C for 30 s, 50°C for 1 min and 72°C for 1.5 min, and final extension at 72°C for 10 min. DNA sequencing was performed at Beijing Genomics Institute, and the sequences were deposited in GenBank^4^ (Table 1). BioEdit v.7.0.5.3 (Hall, 1999) and Geneious Basic v.11.1.15 (Kearse et al., 2012) were used to review the chromatograms and for contig assembly.

**Table 1:** Species and sequences used in the phylogenetic analyses.

Species	Specimen No.	Locality	GenBank accession no.		References
			ITS	28S	
*Coniophora arida*	FP-104367	United States	GU187510	GU187573	Binder et al. (2010)
*Coniophora arida*	CBS:109.40	United States	MH856052	MH867547	Vu et al. (2018)
*Coniophora arida*	Ca30M-20Aa	Poland	MT939247	—	Pusz et al. (2020)
*Coniophora arida*	He 4658	China	MG763875	MH476322	Zhao et al. (2018)
***Coniophora beijingensis***	He 6635*	**China**	**MW192496**	**MW191807**	**Present study**
***Coniophora beijingensis***	**He 6920**	**China**	**MW192497**	**MW191808**	**Present study**
***Coniophora beijingensis***	**He 6926**	**China**	**MW192498**	**MW191809**	**Present study**
*Coniophora cerebella*	HK ‘8’	United States	GU187513	GU187569	Binder et al. (2010)
*Coniophora cystidiophora*	CBS 153.33	Germany	MH855390	MH866840	Vu et al. (2018)
*Coniophora eremophila*	MA-Fungi 86371	Cape Verde	HG326617	—	Unpublished
*Coniophora eremophila*	MA-Fungi 86372	Cape Verde	HG326618	—	Unpublished
*Coniophora eremophila*	Gilbertson 10925	United States	HF921465	—	Unpublished
*Coniophora fusispora*	MA-Fungi 57734	France	HF921467	—	Unpublished
*Coniophora fusispora*	He 6777	China	MW192495	MW191806	Present study
*Coniophora hanoiensis*	He 5197	Vietnam	MG763873	—	Zhao et al. (2018)
*Coniophora hanoiensis*	He 5202	Vietnam	MG763874	MH476323	Zhao et al. (2018)
*Coniophora hanoiensis*	Ryvarden 24995	Zimbabwe	HF921468	—	Unpublished
*Coniophora marmorata*	CLZhao 3577	China	MK268870	—	Unpublished
*Coniophora marmorata*	MUCL 31667	Belgium	GU187515	GU187571	Binder et al. (2010)
*Coniophora marmorata*	P 307	United Kingdom	AJ518880	AJ583427	Moreth and Schmidt (2005); Schmidt et al. (2002)
*Coniophora merulioides*	CBS 152.35	Germany	MH855612	MH867121	Vu et al. (2018)
*Coniophora mollis*	Prem 36877	South Africa	HF921469	—	Unpublished
*Coniophora olivacea*	He 6100	China	OM100743	OM083975	Present study
*Coniophora olivacea*	He 6111	China	OM100744	OM083976	Present study
*Coniophora olivacea*	FP-104386	United States	GU187516	GU187572	Binder et al. (2010)
*Coniophora prasinoides*	MA-Fungi 19417	United States	AJ419197	—	Martín and Raidl (2002)
*Coniophora prasinoides*	Bochum 178449	Germany	HF912261	—	Unpublished
*Coniophora prasinoides*	FP-105969	United States	GU187519	GU187576	Binder et al. (2010)
*Coniophora puteana*	He 2909	China	MG763876	MH476324	Zhao et al. (2018)
*Coniophora puteana*	MUCL 1000	Germany	GU187521	GU187578	Binder et al. (2010)
*Coniophora puteana*	CBS 148.32	Germany	MH855248	MH866701	Vu et al. (2018)
*Coniophora* sp.	UFMGCB 1045	Brazil	FJ605253	—	Unpublished
*Coniophora* sp.	Olrim 361	Sweden	AY781253	—	Vasiliauskas et al. (2005)
*Gloeophyllum sepiarium*	Cui 9237	China	KC782725	KC782735	He et al. (2014)
*Tapinella panuoides*	He 6538	China	MW192499	MW191810	Present study
*Veluticeps abietina*	UC2022888	United States	KP814229	—	Unpublished
*Veluticeps abietina*	UC2022895	United States	KP814230	—	Unpublished
*Veluticeps abietina*	A10-8963	Switzerland	KT943929	—	Unpublished
*Veluticeps abietina*	KHL 12474	Sweden	EU118619	EU118619	Larsson (2007)
*Veluticeps africana*	CBS 403.83	Gabon	MH861619	MH873336	Vu et al. (2018)
*Veluticeps ambigua*	CBS 126033	United States	MH863895	MH875356	Vu et al., 2018
*Veluticeps ambigua*	He 821	China	JQ844472	—	He and Li (2013)
*Veluticeps ambigua*	He 824	China	JQ844473	KJ010080	He and Li (2013) and Yang et al. (2016)
*Veluticeps berkeleyana*	RLG-7116-Sp	United States	KP670408	KP670411	Yang et al. (2016)
*Veluticeps berkeleyana*	HHB-8594-Sp	United States	HM536099	HM536081	Garcia-Sandoval et al. (2011)
*Veluticeps berkeleyana*	He 5261	Vietnam	MW192500	MW191811	Present study
*Veluticeps fasciculata*	Dai 6237	China	KP670406	KP670410	Yang et al. (2016)
*Veluticeps fasciculata*	Dai 14900	China	KP670404	KP670409	Yang et al. (2016)
*Veluticeps fasciculata*	Dai 15092	China	KP670405	**—**	Yang et al. (2016)
*Veluticeps fasciculata*	He 2321	China	KT750961	KT750962	Yang et al. (2016)
*Veluticeps fasciculata*	He 2022	China	KJ010077	KJ010079	Yang et al. (2016)
*Veluticeps fimbriata*	He 20120920-8	China	KP670402	**—**	Yang et al. (2016)
*Veluticeps fimbriata*	L-10628-Sp	United States	HM536100	HM536083	Garcia-Sandoval et al. (2011)
*Veluticeps microspora*	He 651	China	JQ844469	**—**	He and Li (2013)
*Veluticeps microspora*	He 656	China	JQ844470	**—**	He and Li (2013)
*Veluticeps microspora*	He 670	China	JQ844471	**—**	He and Li (2013)
*Veluticeps microspora*	He 5772	Sri Lanka	MW192501	MW191812	Present study
*Veluticeps nakasoneae*	He 585	China	KT944285	**—**	Yang and He (2016)
*Veluticeps nakasoneae*	He 20140720-2	China	KT944286	**—**	Yang and He (2016)
**“*Veluticeps ambigua*”**	**HE21074**	**China**	**KC505560**	**—**	**Unpublished**
**“*Veluticeps berkeleyana*”**	**A6**	**China**	**KC414241**	**—**	**Unpublished**
***Veluticeps subfasciculata***	**He 2046**	**China**	**MW192503**	**MW191814**	**Present study**
***Veluticeps subfasciculata***	He 2979*	**China**	**MW192502**	**MW191813**	**Present study**

New species are set in bold with type specimens indicated with an asterisk (*).

### Phylogenetic analyses

Two separate datasets of concatenated ITS-28S sequences of the *Coniophora* and *Veluticeps* were analyzed. *Tapinella panuoides* (Fr.) E.-J. Gilbert and *Gloeophyllum sepiarium* (Wulfen) P. Karst. were selected as the outgroup for the two datasets, respectively. The ITS and 28S sequences were aligned separately using MAFFT v.7^5^ (Katoh et al., 2017) with the G-INS-I iterative refinement algorithm and optimized manually in BioEdit v.7.0.5.3. Then, the separate alignments were concatenated using Mesquite v.3.5.1 (Maddison and Maddison, 2018).

Maximum parsimony (MP), maximum likelihood (ML) analyses and Bayesian inference (BI) were carried out by using PAUP* v.4.0b10 (Swofford, 2002), RAxML v.8.2.10 (Stamatakis, 2014) and MrBayes 3.2.6 (Ronquist et al., 2012), respectively. In MP analysis, trees were generated using 100 replicates of random stepwise addition of sequence and tree-bisection reconnection (TBR) branch-swapping algorithm with all characters given equal weight. Branch supports for all parsimony analyses were estimated by performing 1,000 bootstrap replicates with a heuristic search of 10 random-addition replicates for each bootstrap replicate. In ML analysis, statistical support values were obtained using rapid bootstrapping with 1,000 replicates, with default settings used for other parameters. For BI, the best-fit substitution model was estimated with jModeltest v.2.17 (Darriba et al., 2012). Four Markov chains were run for 2 million generations for the *Coniophora* dataset and 1 million generations for the *Veluticeps* dataset until the split deviation frequency value was lower than 0.01. Trees were sampled every 100th generation. The first quarter of the trees, which represented the burn-in phase of the analyses, were discarded, and the remaining trees were used to calculate posterior probabilities (BPP) in the majority rule consensus tree.

## Results

### Phylogenetic analyses

**Figure 1:**
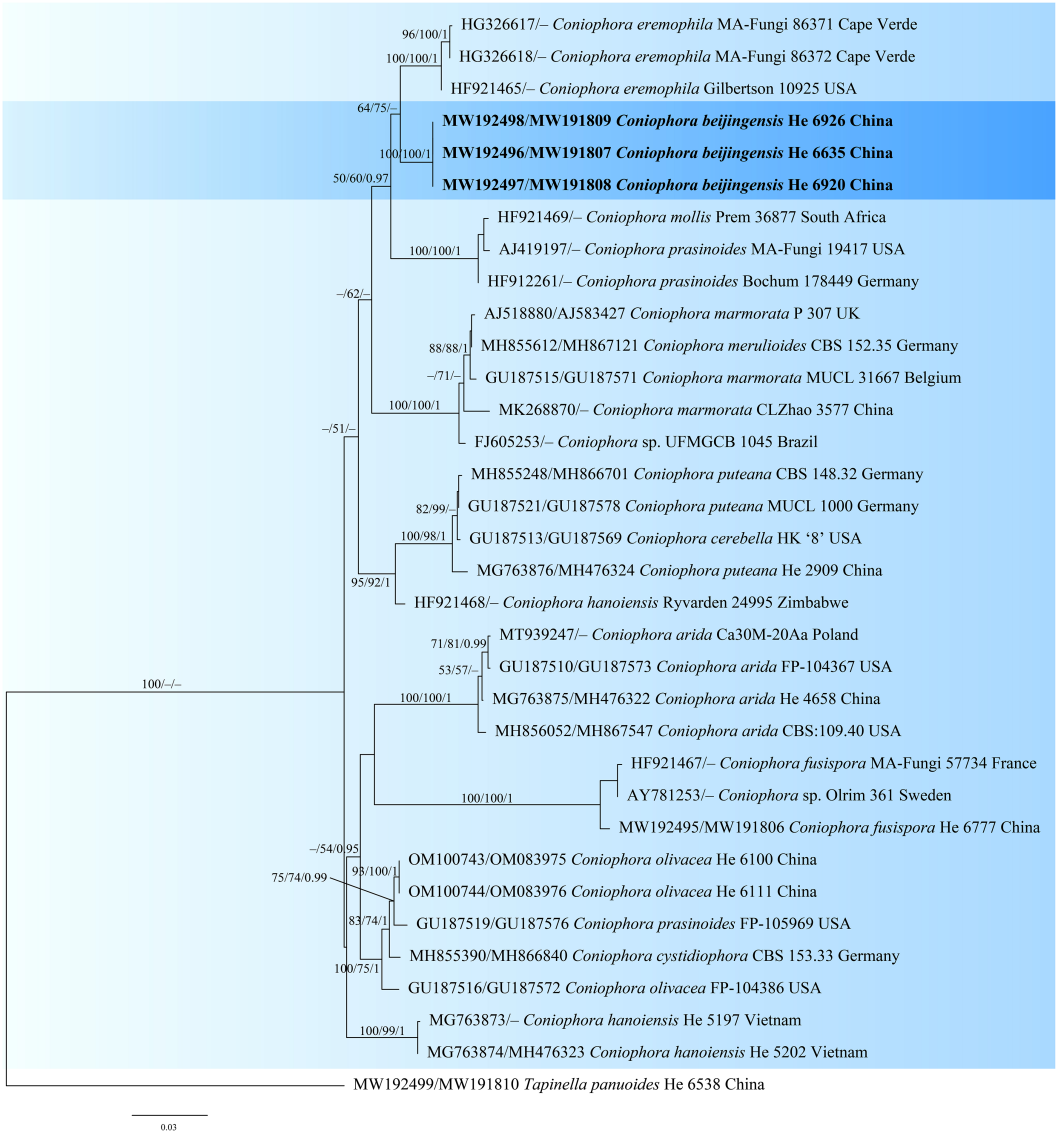
Phylogenetic tree obtained from ML analysis of the ITS-28S sequences of *Coniophora*. Branches are labelled with parsimony bootstrap values (≥50%, first), likelihood bootstrap values (≥50%, second) and Bayesian posterior probabilities (≥0.95, third). New species are set in bold and highlighted.

**Figure 2:**
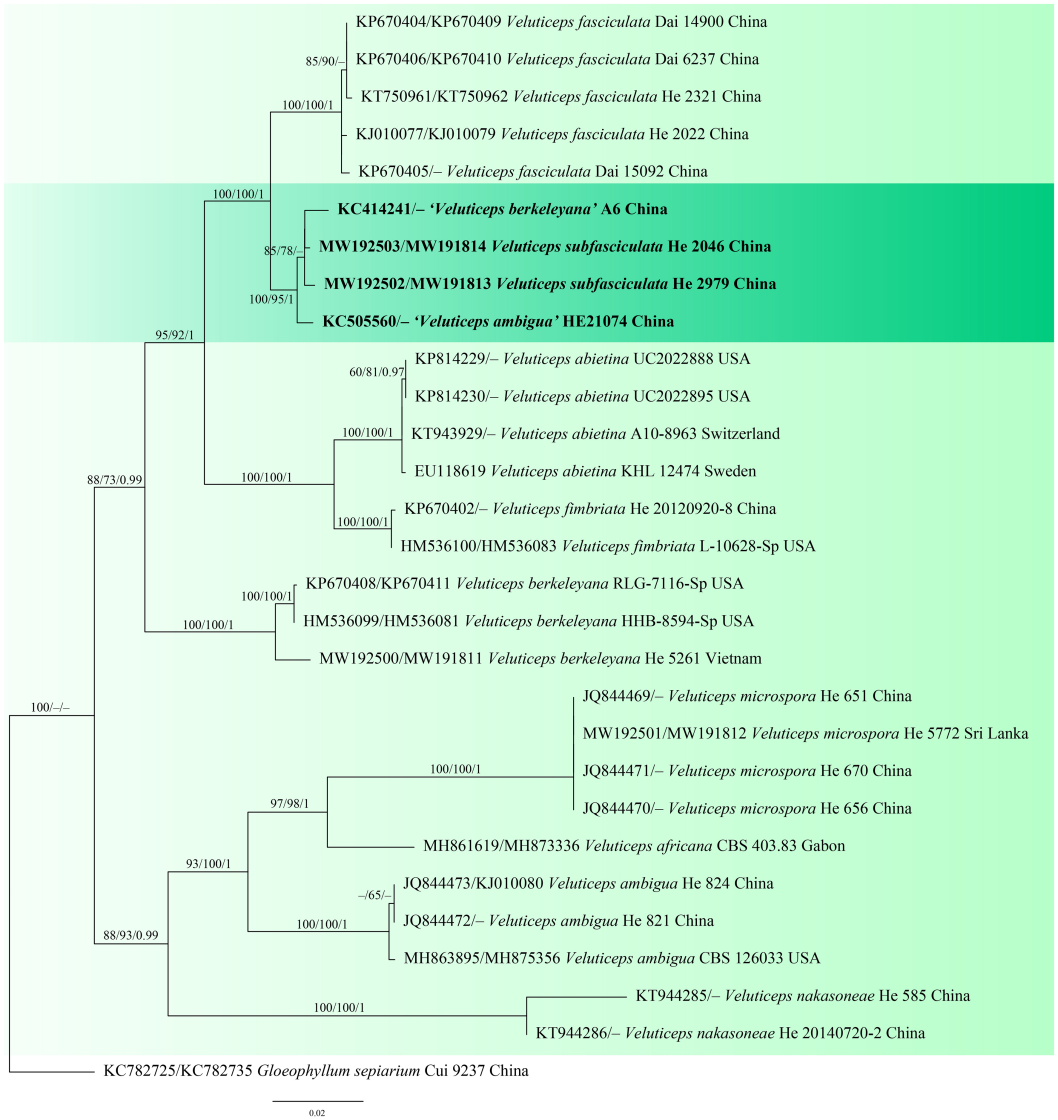
Phylogenetic tree obtained from ML analysis of the ITS-28S sequences of *Veluticeps*. Branches are labelled with parsimony bootstrap values (≥50%, first), likelihood bootstrap values (≥50%, second) and Bayesian posterior probabilities (≥0.95, third). New species are set in bold and highlighted.

The *Coniophora* dataset contained 34 ITS and 21 28S sequences from 34 samples representing 15 ingroup taxa and the outgroup (Table 1). The aligned length was 2040 characters, of which 269 were parsimony informative. MP analysis yielded eight equally parsimonious trees (TL = 784, CI = 0.699, RI = 0.834, RC = 0.583, HI = 0.301). The *Veluticeps* dataset composed of 29 ITS and 16 28S sequences from 29 samples representing 9 ingroup taxa and the outgroup (Table 1). This dataset had an aligned length of 1,884 characters, of which 277 characters were parsimony informative. MP analysis yielded two equally parsimonious tree (TL = 638, CI = 0.743, RI = 0.893, RC = 0.663, HI = 0.257). jModelTest suggested GTR + I + G and GTR + G as the best-fit models of nucleotide evolution for the *Coniophora* and *Veluticeps* datasets, respectively. The average standard deviation of split frequencies of BI was 0.005158 (for the *Coniophora* dataset) and 0.006744 (for the *Veluticeps* dataset) at the end of the run. MP and BI analyses resulted in almost identical tree topologies compared to the ML analysis for both datasets. The ML trees of the two genera are shown in Figures 1, 2, respectively, with the parsimony bootstrap values (≥50%, first value), likelihood bootstrap values (≥50%, second value) and Bayesian posterior probabilities (≥0.95, third value) labelled along the branches.

In the trees, the two new species *Coniophora beijingensis* and *Veluticeps subfasciculata* formed distinct lineages. *Coniophora beijingensis* is sister to *C. eremophila* Lindsey & Gilb. and *C. prasinoides* (Bourdot & Galzin) Bourdot & Galzin, while *Veluticeps subfasciculata* is closely related to *V. fasciculata* Jiao Yang & S.H. He.

### Taxonomy

**Figure 3:**
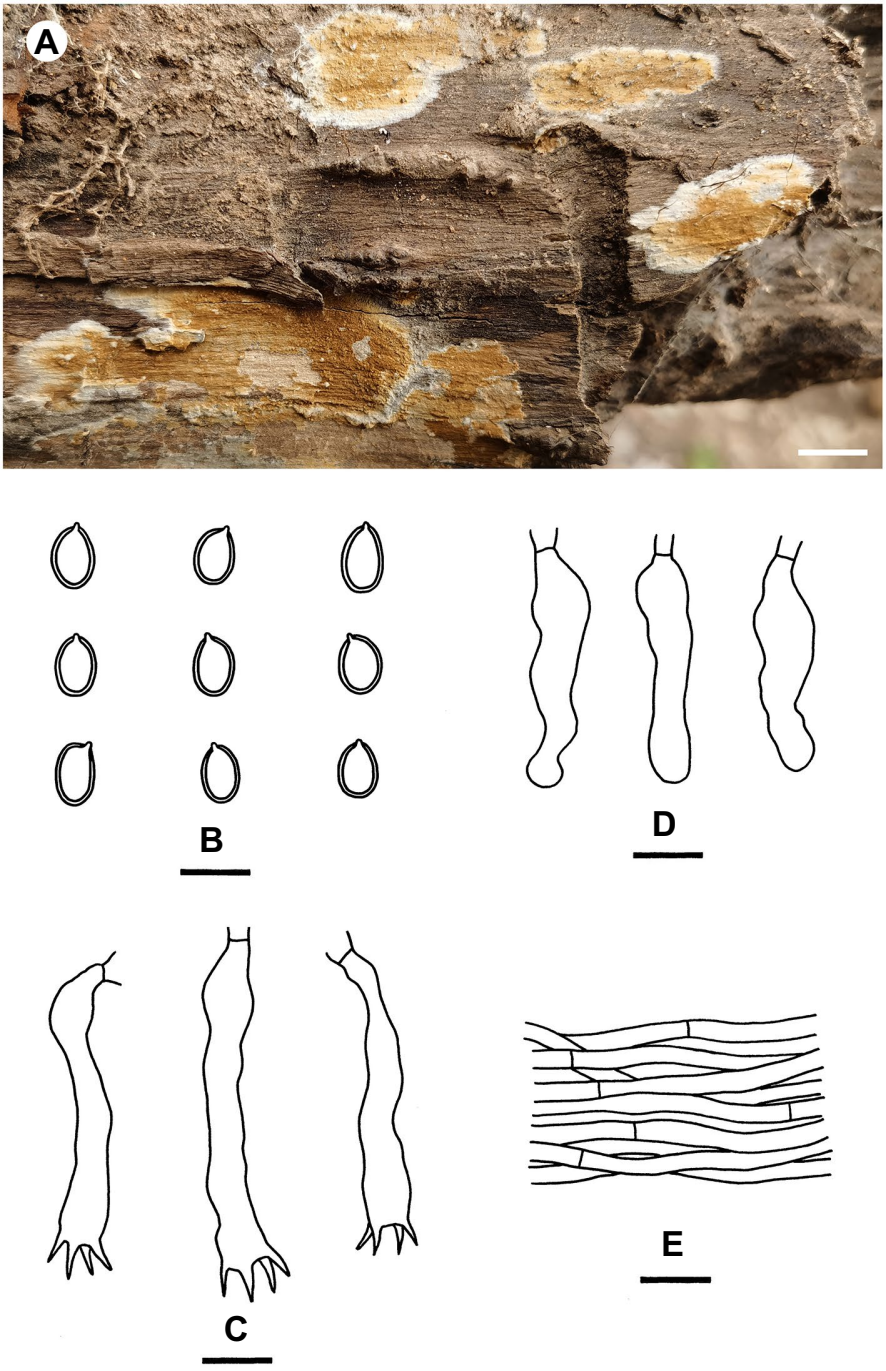
*Coniophora beijingensis* (from the holotype He 6635; scale bars: a = 1 cm; b–e = 10 μm). **(A)** Basidiomes; **(B)** Basidiospores; **(C)** Basidia; **(D)** Basidioles; **(E)** Hyphae from subiculum.

***Coniophora beijingensis*** Yue Li & S.H. He, **sp. nov.** Figure 3

MycoBank: MB847029

Type: China, Beijing, Shijingshan District, Badachu Park, on fallen angiosperm trunk, 30 July 2020, He 6635 (BJFC 033583, holotype).

Etymology: Refers to the type locality in Beijing, China.

Fruiting body: Basidiomes annual, resupinate, effused, closely adnate, inseparable from substrate, membranaceous, first as small patches, later confluent up to 15 cm long, 4 cm wide, 350 μm thick in section. Hymenophore smooth to slightly tuberculate, brownish orange [6C(4–8)] to light brown [6D(4–8)], turning reddish brown in KOH, not cracked; margin thinning out, adnate, without hyphal strands, white to pale yellow, distinct, up to 0.4 cm wide. Context pale yellow.

Microscopic structures: Hyphal system monomitic; all hyphae without clamps. Subiculum distinct, usually with numerous crystals; hyphae colorless, thin- to slightly thick-walled, smooth, rarely branched, moderately septate, tightly interwoven, usually collapsed, 2–5 μm in diam. Subhymenium thickening, composed of collapsed hymenial elements, basidiospores and usually crystals; hyphae colorless, thin-walled, smooth, rarely branched, moderately septate, densely interwoven, slightly agglutinated, 1.5–4 μm in diam. Cystidia absent. Basidia clavate to subcylindrical, colorless, thin-walled, smooth, with four sterigmata and a basal septum, 15–35 × 4–7 μm; basidioles in shape similar to basidia but slightly smaller. Basidiospores ellipsoid to ovoid, with a distinct apiculus, pale yellow, distinctly thick-walled, smooth, strongly dextrinoid in Melzer’s reagent, cyanophilous (6.7–) 7–8.6 (−9.7) × 4.5–6 μm, L = 7.5 μm, W = 5.2 μm, Q = 1.37–1.57 (*n* = 60/2).

Additional specimens examined: China, Beijing, Changping District, Mangshan Forest Park, on dead angiosperm branch, 1 September 2020, He 6920 (BJFC 033869); on fallen decorticated trunk of *Pinus*, 1 September 2020, He 6926 (BJFC 033875); Haidian District, Baiwangshan Forest Park, on dead *Platycladus orientalis* tree, 20 August 2022, He 7732 (BJFC 038868); on dead angiosperm branch, 25 August 2022, He 7754 (BJFC 038890); Xishan Forest Park, on fallen angiosperm trunk, 13 August 2022, He 7685 (BJFC 038821); Yangtaishan Forest Park, on fallen trunk of *Syringa*, 19 August 2022, He 7722 (BJFC 038858).

Notes: *Coniophora beijingensis* is characterized by possessing a monomitic hyphal system with colorless hyphae and relatively small pale yellow basidiospores. *Coniophora arida* (Fr.) P. Karst. is similar to *C. beijingensis* in the basidiomes configurations but differs in having larger basidiospores (10.4–13 μm × 6–8 μm, Ginns, 1982). *Coniophora prasinoides* (Bourdot & Galzin) Bourdot & Galzin is similar to *C. beijingensis* by sharing the colorless hyphae and small basidiospores (7.4–10 μm × 4.3–6.9 μm) but differs in having hyphal strands, scattered verticillate clamps on hyphae and weakly dextrinoid basidiospores (Ginns, 1982). In the phylogenetic tree (Figure 1), *C. beijingensis* formed a distinct lineage sister to *C. eremophila*, which differs in having hyphal strands, loosely interwoven hyphae and larger basidiospores (9.6–11 μm × 6.8–7.6 μm, Ginns, 1982).

**Figure 4:**
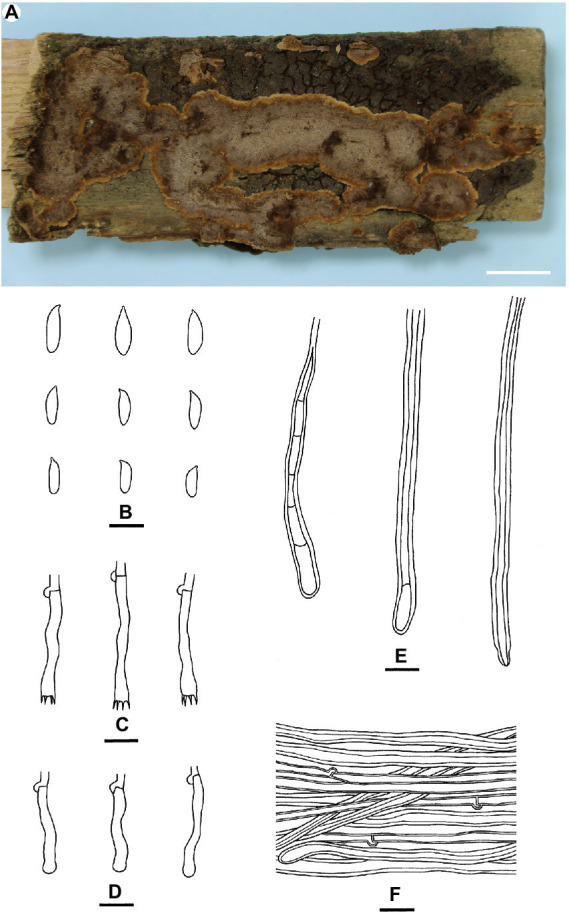
*Veluticeps subfasciculata* (from the holotype He 2979; scale bars: a = 1 cm; b–*f* = 10 μm). **(A)** Basidiomes; **(B)** Basidiospores; **(C)** Basidia; **(D)** Basidioles; **(E)** Skeletocystidia; **(F)** Hyphae from subiculum.

***Veluticeps subfasciculata*** Yue Li & S.H. He, **sp. nov.** Figure 4

MycoBank: MB847030

Type: China, Guizhou Province, Guiyang, Huaxi Park, on fallen trunk of *Cupressus*, 25 August 2010, He 2979 (BJFC 022046, holotype).

Etymology: Refers to the morphological similarity and close phylogenetic relationship to *V. fasciculata* Jiao Yang & S.H. He.

Fruiting body: Basidiomes perennial, resupinate to effused-reflexed effused, adnate, separable from substrate, coriaceous to soft corky, first as small patches, later confluent. Reflexed parts protruding up to 2 cm long, 4 cm wide, up to 0.4 cm thick at base; abhymenial surface glabrous, brown [6E(4–8)] to dark brown [6F(4–8)], sulcate and zonate; margin blunt, paler than abhymenial surface. Hymenophore uneven, colliculose to slightly grandinioid with protruding hyphal pegs, greyish brown [6(D–E)3], light brown [6D(4–6)] to brown [6E(4–6)], turning black in KOH, not cracked or deeply cracked with age; margin thinning out, distinct, velvety, adnate or slightly elevated with age, paler than hymenophore, brownish orange [6C(3–5)] to light brown [6D(4–6)], up to 0.1 cm wide. Context light brown.

Microscopic structures: Hyphal system monomitic; generative hyphae with clamps. Abhymenial tomentum usually present, composed of loosely interwoven brown hyphae. Cutis present in aged parts, composed of densely interwoven brown hyphae. Subiculum distinct, thick, composed of more or less horizontally arranged hyphae; subicular hyphae of two types (1) generative hyphae colorless, slightly thick-walled, smooth, moderately septate and branched, 2–4 μm in diam; (2) sclerified hyphae pale yellow to golden yellow, distinctly thick-walled, smooth, with rare clamps, usually with secondary septa, unbranched; transitions of the two kinds of hyphae present. Subhymenium thickening, composed of more or less vertically arranged hyphae, collapsed hymenial elements and skeletocystidia; subhymenial hyphae colorless, slightly thick-walled, smooth, 2–3.5 μm in diam. Skeletocystidia hyphoid, tubular to clavate, originated from subhymenium or subiculum, single or usually with several to many fasciculate together forming the hyphal pegs, colorless, yellow to yellowish brown, distinctly thick-walled, smooth or encrusted with crystals with age, some with one to several secondary septa, with a basal clamp, up to 150 μm long, 3–8 μm wide, protruding up to 80 μm above the hymenium. Basidia subclavate to subcylindrical, colorless, thin-walled, smooth, with four sterigmata and a basal clamp, 20–35 μm × 4–6 μm; basidioles numerous, in shape similar to basidia, but slightly smaller. Basidiospores subcylindrical to subfusiform, colorless, thin-walled, smooth, negative in Melzer’s reagent, acyanophilous, 8–11 × 2.5–3.5 (−4) μm, L = 9.7 μm, W = 3.1 μm, Q = 3–3.2 (*n* = 90/3).

Additional specimens examined: China, Sichuan Province, Jiange County, Cuiyunlang scenic spot, on fallen trunk of *Cupressus*, 18 June 2011, He 2046 (BJFC 016644); Jianmenguan scenic spot, on trunk of dead *Cupressus*, 9 November 2018, Dai 19384 (BJFC 027852).

Notes: *Veluticeps subfasciculata* is characterized by the resupinate to effused-reflexed basidiomes with a colliculose hymenophore, nodose-septate generative hyphae, fasciculate skeletocystidia and growing on trunks of *Cupressus* in southwestern China. *Veluticeps fasciculata* is similar to *V. subfasciculata* by sharing the colliculose hymenophore, nodose-septate generative hyphae and fasciculate skeletocystidia but differs in having longer basidia (30–65 μm) and growing on *Cunninghamia* and *Cryptomeria* in central and southeastern China (Yang et al., 2016). *Veluticeps berkeleyana* is also similar to *V. subfasciculata* by sharing fasciculate cystidia and nodose-septate generative hyphae but differs in having larger basidiospores (12–14.5 μm × 4–5 μm) and growing on *Pinus* (Nakasone, 1990). In the phylogenetic tree (Figure 2), two samples from GenBank, “*V. berkeleyana* isolate A6 (KC414241)” and “*V. ambigua* isolate HE21074 (KC505560)” both from Sichuan Province, southwestern China clustered with the type specimens with strong support values and formed the *V. subfasciculata* lineage, which is closely related to *V. fasciculata*.

## Discussion

Although the brown-rot corticioid fungi only account for a small portion of the wood-decaying fungi in nature, they also play important roles in the cycling of substances and renewal of ecosystem. Our present and previous studies (He and Li, 2013; Yang et al., 2016; Yang and He, 2016) indicated that the species diversity of this group of fungi in China is rich and needs more investigations and studies. Future works should be emphasized on the special habitats and hosts, for example, the gymnosperm trees in areas of high altitudes. With more specimens collected and sequenced, the number of this group fungi in China will be largely increased in future.

*Coniophora* and *Veluticeps* are two common brown-rot corticoid genera in China. Morphologically, both genera have brown basidiomes, but *Coniophora* in Boletales can be easily distinguished from *Veluticeps* in Gloeophyllales by having yellowish-brown, thick-walled basidiospores. *Hymenochaete* Lév. in Hymenochaetales is similar to *Coniophora* and *Veluticeps* by sharing resupinate brown basidiomes, but differs in having characteristic brown setae.

*Coniophora fusispora*, a characteristic and widely distributed species, is reported from China for the first time based on two specimens collected on the bases of living *Pinus* tree in Beijing. The species numbers of *Coniophora* and *Veluticeps* in China have now become to seven and six with two new species and a new Chinese record reported above. Herein, we present two identification keys to all species of the two genera as follows.

### A key to *Coniophora* species in China

**Table:** 

1. Basidiospores subfusiform	*C. fusispora*
1. Basidiospores ellipsoid, broadly ellipsoid to ovoid	2
2. With large, brown, septate cystidia	*C. olivacea*
2. Cystidia absent	3
3. Hyphal strands present, hyphal system dimitic	*C. marmorata*
3. Hyphal strands absent, hyphal system monomitic	4
4. Context brown to dark brown, hyphae brown	*C. matsuzawae*
4. Context pale yellow, hyphae colorless	5
5. Basidiopores < 9 μm long, <6 μm wide	*C. beijingensis*
5. Basidiopores > 9 μm long, >6 μm wide	6
6. Basidiomes thin, adnate, brittle	*C. arida*
6. Basidiomes thick, separable, membranaceous	*C. puteana*

### A key to *Veluticeps* species in China

**Table:** 

1. Generative hyphae simple-septate	2
1. Generative hyphae nodose-septate	4
2. Brown dendrophyhidia present; basidiospores 18–22 μm × 5.8–7 μm	*V. nakasoneae*
2. Dendrophyhidia absent; basidiospores up to 16 μm × 4 μm	3
3. Basidiospores 7–9 × 2.5–3 μm	*V. microspora*
3. Basidiospores 12–16 × 3.2–4 μm	*V. ambigua*
4. Hymenophore smooth, without hyphal pegs; on Abies or Picea	*V. fimbriata*
4. Hymenophore developing hyphal pegs; on other hosts	5
5. On *Cunninghamia* and *Cryptomeria* in central and southeastern China	*V. fasciculata*
5. On *Cupressus* in southwestern China	*V. subfasciculata*

## Data availability statement

The datasets presented in this study can be found in online repositories. The names of the repository/repositories and accession number(s) can be found at: https://www.ncbi.nlm.nih.gov/genbank/, MW192496–MW192498; MW191807–MW191809; MW192503; MW192502; MW191814; and MW191813.

## Author contributions

YL performed the phylogenetic analyses, did most of the measurements, descriptions and illustrations, and wrote the draft of the manuscript. S-HH designed the research, collected most of the specimens, and revised the manuscript. All authors contributed to the article and approved the submitted version.

## Funding

Financial support was provided by the National Natural Science Foundation of China (nos. 32270014 and 31750001).

## Conflict of interest

The authors declare that the research was conducted in the absence of any commercial or financial relationships that could be construed as a potential conflict of interest.

## Publisher’s note

All claims expressed in this article are solely those of the authors and do not necessarily represent those of their affiliated organizations, or those of the publisher, the editors and the reviewers. Any product that may be evaluated in this article, or claim that may be made by its manufacturer, is not guaranteed or endorsed by the publisher.

## Footnotes

^1^ https://www.indexfungorum.org

^2^ http://sweetgum.nybg.org/science/ih/

^3^ https://sites.duke.edu/vilgalyslab/rdna_primers_for_fungi/

^4^ https://www.ncbi.nlm.nih.gov/

^5^ http://mafft.cbrc.jp/alignment/server/

## Abbreviations

ITS: Internal transcribed spacer
28S: Nuclear ribosomal large subunit
BJFC: Herbarium of Beijing Forestry University, Beijing, China
KOH: 2% (w/v) potassium hydroxide
IKI: Melzer’s reagent
CB: Cotton blue
IKI–: Neither amyloid nor dextrinoid
CB–: Acyanophilous
L: Mean basidiospore length
W: Mean basidiospore width
Q: L/W ratio, n (a/b), number of basidiospores (a) measured from number of specimens (b)
CTAB: Cetyltrimethylammonium bromide
DNA: Deoxyribonucleic acid
PCR: Polymerase chain reaction
MP: Maximum parsimony
ML: Maximum likelihood
BI: Bayesian inference
BPP: Bayesian posterior probability
